# Tensor framelet based iterative image reconstruction algorithm for low-dose multislice helical CT

**DOI:** 10.1371/journal.pone.0210410

**Published:** 2019-01-11

**Authors:** Haewon Nam, Minghao Guo, Hengyong Yu, Keumsil Lee, Ruijiang Li, Bin Han, Lei Xing, Rena Lee, Hao Gao

**Affiliations:** 1 Department of Liberal Arts, Hongik University, Sejong, Republic of Korea; 2 School of Biomedical Engineering, Shanghai Jiao Tong University, Shanghai 200240, China; 3 Department of Electrical and Computer Engineering, University of Massachusetts, Lowell, Massachusetts 01854, United States of America; 4 Department of Radiology, Stanford University, Stanford, California 94305, United States of America; 5 Department of Radiation Oncology, Stanford University, Stanford, California 94305, United States of America; 6 Department of Radiation Oncology, Ewha Womans University, Seoul, Korea; 7 Department of Radiation Oncology, Emory University, Atlanta, GA 30322, United States of America; North Shore Long Island Jewish Health System, UNITED STATES

## Abstract

In this study, we investigate the feasibility of improving the imaging quality for low-dose multislice helical computed tomography (CT) via iterative reconstruction with tensor framelet (TF) regularization. TF based algorithm is a high-order generalization of isotropic total variation regularization. It is implemented on a GPU platform for a fast parallel algorithm of X-ray forward band backward projections, with the flying focal spot into account. The solution algorithm for image reconstruction is based on the alternating direction method of multipliers or the so-called split Bregman method. The proposed method is validated using the experimental data from a Siemens SOMATOM Definition 64-slice helical CT scanner, in comparison with FDK, the Katsevich and the total variation (TV) algorithm. To test the algorithm performance with low-dose data, ACR and Rando phantoms were scanned with different dosages and the data was equally undersampled with various factors. The proposed method is robust for the low-dose data with 25% undersampling factor. Quantitative metrics have demonstrated that the proposed algorithm achieves superior results over other existing methods.

## Introduction

X-ray computed tomography (CT) has been one of the most widely used medical imaging techniques since Hounsfield invented the first commercial medical X-ray machine in 1972 [1]. The Helical CT was first invented by I. Mori [2] in the late 1980s and was developed by W. Kalender [3] in the 1990s. The number of detector rows has been increased to achieve larger volume coverage with a reduced scan time and improved z-resolution. The 8-slice CT system was first introduced in 2000, Siemens SOMATOM Definition scanner has 64-slice rows for up to 128-channel data acquisition, and the Toshiba Aquilion ONE ViSION, which has 320-slice rows for generating 640 slices, was brought out in 2013.

Helical CT reconstruction algorithms can be categorized into two groups: Analytic reconstruction and iterative algorithm. An analytic reconstruction can be sub-divided into exact and approximate reconstruction methods. The Feldkamp-Davis-Kress algorithm (FDK) is a well-known approximate analytic reconstruction algorithm [4] and it can be generalized for helical scan trajectories [5–7]. However, FDK generates helical artifacts due to data insufficiency. A conventional filtered backprojection (FBP) algorithm can be implemented with data interpolation [8] to soften helical artifacts, but this may generate another type of artifact caused by data approximation. In 2002, Katsevich introduced an exact FBP-type reconstruction algorithm based on the PI-line and Tam-Danielsson window [9–11]. Details for the numerical implementation of the Katsevich algorithm are given in [12, 13]. An alternative derivation of the Katsevich algorithm is provided by Chen [14]. Meanwhile, another exact method of backprojection-filtration (BPF) has been developed by Zou and Pan [15], and these ideas have inspired several subsequent exact reconstruction methods [16–20].

Katsevich-type algorithms are based on exact analytic theory, and thus it is sensitive to the noisy projection data. To overcome these noise sensitivity, many researchers have studied iterative reconstruction algorithms [21, 22] by formulating the image reconstruction as an optimization problem based on versatile CT models. The Algebraic Reconstruction Technique (ART) [1, 23] and Simultaneous Algebraic Reconstruction Technique (SART) [24] are two of the most popular methods in the early stage of iterative reconstruction algorithms. Iterative reconstruction algorithms are based on a mathematical minimization which seeks the best approximate solution. They have greater flexibility, and thus are robust against noise. A regularization term, such as Tikhonov or total-variation (TV) regularizer, can be added to the cost function for noise and artifact reduction. Compared with analytic reconstruction algorithms, iterative reconstruction algorithms work well even with insufficient or low-dose data. However a significant disadvantage of iterative reconstruction is its high computational cost, particularly for helical CT scans, which contain a huge amount of data. Thanks to recent advances in computing hardware, iterative reconstruction is emerging for helical CT [21, 22, 25, 26]. Nuyts et al. [22] investigated the superiority of iterative reconstruction compared to non-iterative methods for a helical CT system. They concluded that iterative reconstruction improved the axial resolution. Thibault et al. [21] presented a Bayesian iterative reconstruction algorithm for multislice helical data, they showed improved high contrast spatial resolution and signal-to-noise ratio relative to analytic methods. Yu and Zeng [26] developed a TV-based iterative algorithm and applied it to a limited-angle inverse helical CBCT reconstruction for C-arm system.

In this article, we propose an iterative reconstruction algorithm to improve multi-slice helical CT based on tensor framelet (TF) [27, 28] regularization. The method belongs to a sparsity-regularized model-based iterative reconstruction, which is inspired by compressive sensing [29, 30]. To name a few: Sidky et al. develops a TV-based iterative method for sparse-view and limited-angle reconstruction [31]; Chen et al. proposes the so-called PICCS method for the dynamic CT problem [32]; Yu and Wang studies the sparsity-regularized method for interior tomography [33]; Gao et al. proposes a rank-sparsity decomposition method for dynamic CT [34] and spectral CT [35]; Jia et al. studies tight frame regularization for cone-beam CT image reconstruction [36]; Xu et al. develops a dictionary learning-based image reconstruction method for dose reduction [37].

This paper is organized as follows: Section II provides the materials and method details, including the minimization problem, TF regularization, optimization algorithm for iterative multislice helical CT image reconstruction, and experimental details. Section III presents the validation of the proposed method for low-dose multislice helical CT in comparison with FDK, the Katsevich and TF algorithms, with low-dose and sparse-view data. Section IV summarizes this work.

## Materials and methods

### Minimization problem

The mathematical formulation of an iterative CT reconstruction can be expressed by a least-square minimization problem as

$$\begin{array}{c} \text{x}=\arg\min_{\text{x}}\frac{1}{2}{∥\text{Ax}-\text{y}∥}_{2}^{2}+\lambda R\left(\text{x}\right), \end{array}$$ (1)

where **x** is the three-dimensional image to be reconstructed with given projection data **y** and the projection matrix **A**. The first term indicates the data fidelity in the *L*^2^-norm. The second term consists of *R*(**x**) as a regularization function with regularization parameter λ. For example, the TV norm is a popular regularization choice for sparsity-based CT image reconstruction [31, 32].

In this paper, we solve Eq (1) with the given data **y** from the multislice helical CT system. The projection matrix **A** contains the helical geometry with the flying focal spot [38]. For the forward projection **A** and its adjoint **A**^*T*^, parallelized algorithms with an infinitely narrow beam are used with GPU implementation [39].

#### Tensor framelet regularization

Consider a 3D image **x** as a tensor,

$$\begin{array}{c} \text{x}=\{x_{ijk},i\leq N_{x},\;j\leq N_{y},\;k\leq N_{z}\} \end{array}$$

where *x*_*ijk*_ is the (*i*, *j*, *k*)-th voxel in three-dimensional image space, *N*_*x*_, *N*_*y*_, and *N*_*z*_ are the number of voxels in the *x*, *y* and *z*–axis respectively. We define **x**_*x*_, **x**_*y*_, and **x**_*z*_ as 1D unfolded matrices of **x** along the *x*, *y*, and *z*–axes, respectively. The TF transform is constructed using the standard 1*D* framelet transform [40], e.g., the 1D piecewise linear tight frame with the following refinement masks.

$$\omega_{0}=\frac{1}{4}\left[1\;2\;1\right],\;\omega_{1}=\frac{\sqrt{2}}{4}\left[1\;0\;-1\right],\;\omega_{2}=\frac{1}{4}\left[-1\;2\;-1\right].$$

The operator *ω*_0_ is an averaging operator, and the two other operators *ω*_1_ and *ω*_2_ are the first and second differential operators, respectively. Note that *ω*_0_ smoothes the image, while *ω*_1_ and *ω*_2_ enhance the edges. Define

$$\begin{array}{c} {\text{M}}_{j}\text{x}=\frac{1}{\sqrt{3}}[\begin{array}{c} \omega_{j}*{\text{x}}_{x}\\ \omega_{j}*{\text{x}}_{y}\\ \omega_{j}*{\text{x}}_{z} \end{array}],\forall j=0,1,2, \end{array}$$

where * denotes the convolution operator. The TF regularization function **W** and its adjoint **W**^*T*^ are respectively defined as below.

$$\begin{array}{c} \text{Wx}={\left[{\text{M}}_{0}\text{x},\;{\text{M}}_{1}\text{x},\;{\text{M}}_{2}\text{x}\right]}^{T}, \end{array}$$ (2)

and

$$\begin{array}{c} {\text{W}}^{T}\text{y}={\text{M}}_{0}^{T}\left({\text{M}}_{0}\text{x}\right)+{\text{M}}_{1}^{T}\left({\text{M}}_{1}\text{x}\right)+{\text{M}}_{2}^{T}\left({\text{M}}_{2}\text{x}\right),\;\text{for}\;\text{y}=\text{Wx}. \end{array}$$ (3)

The TF norm is defined as λ‖**Wx**‖_1_ = λ_0_‖**M**_0_**x**‖_1_ + λ_1_‖**M**_1_**x**‖_1_ + λ_2_‖**M**_2_**x**‖_1_, where $\|{\text{M}}_{j}\text{x}{\|}_{1}=\sqrt{|\omega_{j}*{\text{x}}_{x}{|}^{2}+|\omega_{j}*{\text{x}}_{y}{|}^{2}+|\omega_{j}*{\text{x}}_{z}{|}^{2}}$, for all *j* = 0, 1, and 2. TF transform **W** is left invertible and **W**^*T*^**W** = *I*, by the simple calculation [27]. If λ_0_ = 0, λ_1_ ≠ 0, and λ_2_ = 0, ‖**Wx**‖_1_ corresponds to the isotropic TV norm of **x**. In other words, TF regularization is a high-order generalization of TV.

The TF transform **W** can be extended to the multilevel by diluting the masks to $\omega_{i}^{l}$ such that

$$\begin{array}{ccc} \omega_{0}^{l} & = & \frac{1}{4}\left[1\;\underset{2^{l}-1}{\underset{︸}{0\cdots 0}}\;2\;\underset{2^{l}-1}{\underset{︸}{0\cdots 0}}\;1\right],\\ \omega_{1}^{l} & = & \frac{\sqrt{2}}{4}\left[1\;\underset{2^{l}-1}{\underset{︸}{0\cdots 0}}\;0\;\underset{2^{l}-1}{\underset{︸}{0\cdots 0}}\;-1\right],\\ \omega_{2}^{l} & = & \frac{1}{4}\left[-1\;\underset{2^{l}-1}{\underset{︸}{0\cdots 0}}\;2\;\underset{2^{l}-1}{\underset{︸}{0\cdots 0}}\;1\right]. \end{array}$$

Similar to the uni-level TF, define ${\text{x}}^{l}=\omega_{0}^{l}*{\text{x}}^{l-1}$, **x**^0^ = **x** and

$$\begin{array}{c} {\text{M}}_{j}^{l}{\text{x}}^{l}=\frac{1}{\sqrt{3}}[\begin{array}{c} \omega_{j}^{l}*{\text{x}}_{x}^{l}\\ \omega_{j}^{l}*{\text{x}}_{y}^{l}\\ \omega_{j}^{l}*{\text{x}}_{z}^{l} \end{array}],\forall j=0,\cdots ,2,\forall l=1,\cdots L. \end{array}$$

Then TF transform **W** with level *L* is

$$\begin{array}{c} \text{W}\text{x}=[\underset{1\text{st}\;\text{level}}{\underset{︸}{{\text{M}}_{1}^{1}{\text{x}}^{1}\;{\text{M}}_{2}^{1}{\text{x}}^{1}}}\;\cdots \;\underset{l-\text{th}\;\text{level}}{\underset{︸}{{\text{M}}_{1}^{l}{\text{x}}^{l}\;{\text{M}}_{2}^{l}{\text{x}}^{l}}}\;\cdots \;\underset{L-\text{th}\;\text{level}}{\underset{︸}{{\text{x}}^{L}\;{\text{M}}_{1}^{L}{\text{x}}^{L}\;{\text{M}}_{2}^{L}{\text{x}}^{L}}}\;], \end{array}$$ (4)

and its adjoint **W**^*T*^ is defined as

$$\begin{array}{c} {\text{W}}^{T}\left(\text{y}\right)={\text{x}}^{L}+\sum_{l=1}^{L}\sum_{j=1}^{2}{\text{M}}_{j}^{l}{\text{x}}^{l},\;\text{for}\;\text{y}=\text{Wx} \end{array}$$ (5)

Similarly, Eqs (4) and (5) are a generalization of TV to multilevel, and it keeps the framelet features such as **W**^*T*^(**Wx**) = **x**. With the TF regularization, Eq (1) becomes

$$\begin{array}{c} \text{x}=\arg\min_{\text{x}}\frac{1}{2}{∥\text{Ax}-\text{y}∥}_{2}^{2}+\lambda {∥\text{Wx}∥}_{1}. \end{array}$$ (6)

The TF regularization term is defined as the isotropic shrinkage TF norm [27]:

$$\begin{array}{c} {\lambda ∥\text{Wx}∥}_{1}=\sum_{l=1}^{L}\sum_{j=1}^{2}\lambda_{l,j}∥{\text{M}}_{j}^{l}{\text{x}}^{l}{∥}_{1}+\lambda_{L,0}{∥{\text{x}}^{L}∥}_{1}. \end{array}$$ (7)

#### Optimization algorithm

The TF regularization (7) is the summation of *L*_1_-norm. To solve the non-differentiable *L*_1_ minimization problem (6), we choose the alternating direction method of multipliers (ADMM) [41] or the so-called Split Bregman method [42]. In general it is difficult to solve the *L*_1_-regularized minimization problem because it has non-differentiable *L*_1_ term. The basic idea of ADMM is to split *L*_1_ and *L*_2_ components by introducing auxiliary variables **d**, and **v**. Eq (6) becomes

$$\begin{array}{c} \text{x}=\arg\min_{\text{x},\;\text{d},\;\text{v}}{\lambda ∥\text{d}∥}_{1}+\frac{1}{2}{∥\text{Ax}-\text{y}∥}_{2}^{2}+\frac{\mu }{2}{∥\text{Wx}-\text{d}+\text{v}∥}_{2}^{2}, \end{array}$$ (8)

which can be split into three steps:

- **Step 1:** $x^{n+1}=\text{arg}\;{\text{min}}_{x}\frac{1}{2}\|Ax-y{\|}^{2}+\frac{\mu }{2}\|Wx-d^{n}+v^{n}{\|}_{2}^{2}$
- **Step 2:** $d^{n+1}=\text{arg}\;{\text{min}}_{d}\|d{\|}_{1}+\frac{\mu }{2}\|Wx^{n+1}-d+v^{n}{\|}_{2}^{2}$
- **Step 3: v**^**n**+**1**^ = **v**^**n**^ + **Wx**^**n**+**1**^ − **d**^**n**+**1**^

Because of the decoupled form, step 1 is the sum of two differentiable *L*_2_-norm terms. Thus, we can efficiently solve it from its optimal condition by the conjugate gradient method. Note that TF is more computationally efficient than TV due to **W**^*T*^**W** = *I*. Step 2 can be solved efficiently using the TF shrinkage formula [28]. Step 3 is in its explicit form, thus it is easy to implement.

### Experiments

#### Data acquisition

The multislice helical CT reconstruction quality was evaluated using the American College of Radiology (ACR) CT accreditation phantom (Data Spectrum Corporation. Model: ECT/DLX/P) and the Rando phantom. Siemens SOMATOM Definition 64-slice helical CT scanner was used to generate the helical CT projection data. Details of the scan parameters for ACR phantom were as follows: Various voltage parameters with effective mAs, CTDI_vol_, and DLP are described in Table 1. For every voltage level, there was a 3.05 *s* scan time, 0.5 *s* gantry rotation time, and 64 * 0.6 mm collimation with z-flying focal spot. The helical pitch is set to be *p* = 1, with 2304 projections per rotation. Image volume resolution is: 2 *mm* slice thickness and 0.9766 × 0.9766 *mm*^2^ axial resolution. The whole image volume has 512 × 512 × 88 voxels. A 21.6 cm inside diameter cylindrical ACR phantom is used. Parameter details for the Rando phantom scan were as follows: 120kV with 350 effective mAs are used. There was a 17 *s* scan time and 20 * 0.6 mm collimation with z-flying focal spot. The helical pitch is set to be *p* = 1, with 4608 projections per rotation. Image volume resolution is: 4 *mm* slice thickness and 0.9766 × 0.9766 *mm*^2^ axial resolution. The whole image volume has 512 × 512 × 53 voxels.

**Table 1:** Scan parameters with different voltages.

voltage (kV)	Effective mAs	CTDI_vol_(32cm) (mGy)	DLP (mGy-cm)
80	178	3.30	66.3
100	165	6.51	130.8
120	161	10.84	217.7
140	153	15.78	316.8

#### Quantitative metrics

To evaluate the performance of the proposed algorithm quantitatively in comparison to FDK and the Katsevich algorithm, four different quantitative metrics are selected. The Universal Quality Index (QUI) measures the intensity similarity between the reconstructed and true images. Image noise is measured by Signal-to-Noise Ratio (SNR) and Contrast-to-Noise Ratio (CNR). These two metrics quantify the noise level of the reconstructed images. The Modulation Transfer Function (MTF) is used to evaluate the resolution of the reconstructed images.

#### Image similarity—Universal Quality Index (UQI)

The Universal Quality Index (UQI) [43] was measured to evaluate the similarity between the reconstructed and true images. We considered the image from the scanner to be the true image. Given the ROI within the reconstructed and true images, the associative mean of the image *μ*, the variance and covariance of *μ* with the true image *μ*_true_ over the ROI are denoted as $\bar{\mu }$, *σ*^2^, and Cov(*μ*, *μ*_true_), respectively. The definition of UQI is given as

$$\begin{array}{c} \text{UQI}=\frac{4\;\text{Cov}(\mu ,\mu_{\text{true}})}{\sigma^{2}+\sigma_{\text{true}}^{2}}\frac{\bar{\mu }\cdot {\bar{\mu }}_{\text{true}}}{{\bar{\mu }}^{2}+{\bar{\mu }}_{\text{true}}^{2}}. \end{array}$$

The UQI measures the intensity similarity between two images, and its value ranges [0, 1]. A UQI value close to 1 indicates a better level of similarity between the reconstructed and true images. We chose two ROIs: The whole ACR phantom body on slices 10 and 50. We calculated the UQI scores for all three methods under comparison.

#### Image noise—SNR and CNR

To evaluate the quantitative noise level of the reconstructed images, we chose two different metrics, SNR and CNR. The definitions are as follows.

$$\begin{array}{c} \text{SNR}=\frac{{\bar{\mu }}_{\text{ROI}}}{\sigma_{\text{ROI}}} \end{array}$$

$$\begin{array}{c} \text{CNR}=\frac{|{\bar{\mu }}_{\text{ROI}}-{\bar{\mu }}_{{\text{ROI}}_{\text{air}}}|}{\sqrt{\sigma_{\text{ROI}}^{2}+\sigma_{{\text{ROI}}_{\text{air}}}^{2}}} \end{array}$$

where *σ*_ROI_ and $\sigma_{{\text{ROI}}_{\text{air}}}$ refer to the standard deviations and ${\bar{\mu }}_{\text{ROI}}$ and ${\bar{\mu }}_{{\text{ROI}}_{\text{air}}}$ refer to the mean pixel value in a ROI inside and the background of the phantom, respectively. We chose four Regions Of Interest (ROI) to compare the reconstructed images from all three methods with that from the scanner. For convenience, the CT numbers are normalized with 1 as the maximum.

#### Image resolution—MTF

**Fig 1:**
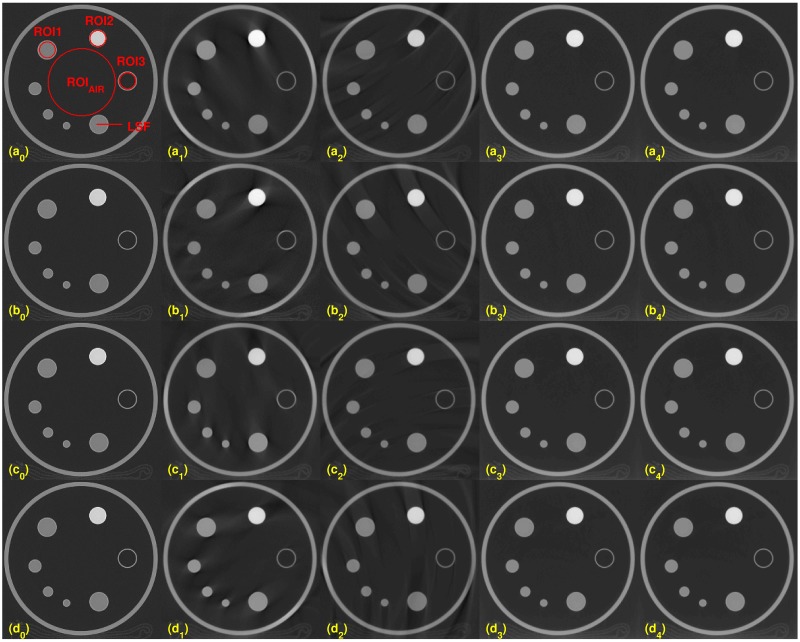
Illustrated reconstructed images with varying kVs on the slice number 10. (a_0_): Image from the scanner. Red circles indicate ROI’s: ROI1, ROI2, ROI3 and ROI_*AIR*_. The red line is used to compute the LSF and MTF. Each row has reconstructed images at different kVs, (a_*j*_): 80kV, (b_*j*_): 100kV, (c_*j*_): 120kV, and (d_*j*_): 140 kV, for all *j* = 0 ⋯ 4. Each column has reconstructed images from different reconstruction algorithms: (X_0_): scanner, (X_1_): FDK, (X_2_): Katsevich, (X_3_): TV, and (X_4_): TF for all letters X = a, b, c or d.

The Modulation Transfer Function (MTF) [43, 44] is calculated to measure resolution of the reconstructed images. An Edge Spread Function (ESF) was obtained along the profile of the red line on Fig 1. The Line Spread Function (LSF) was achieved by differentiating the ESF. The MTF was obtained from the Fourier transformation of the LSF. Normalization was performed as MTF(0) = 1.

## Results

### Evaluations with low-dose data

**Fig 2:**
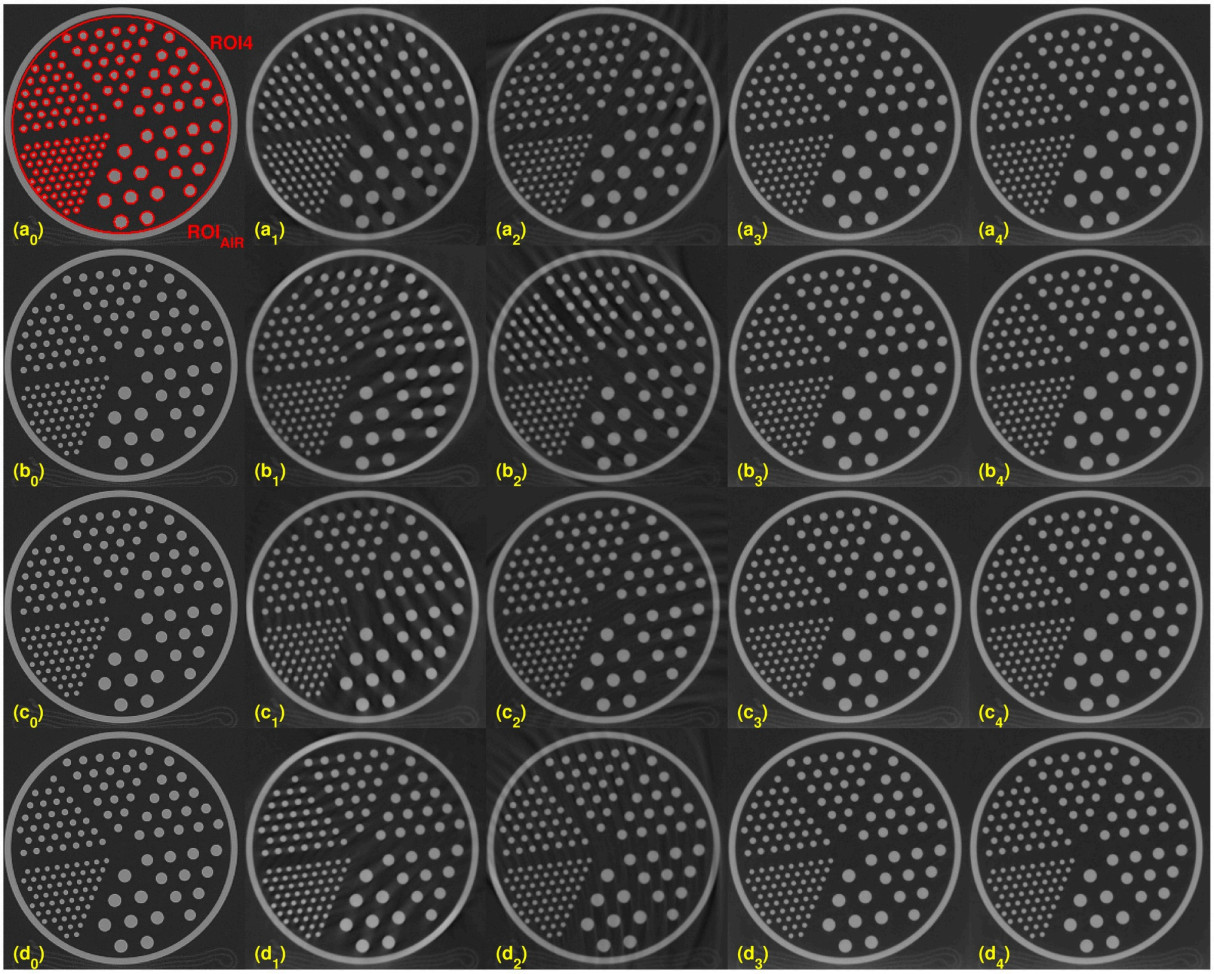
Comparison of the reconstruction algorithms with varying kVs on the 50-th slice. ROI4 is the set of the interiors of the small red circles. ROI_*AIR*_ is defined as the air part inside the phantom on slice 50-th. Each row has reconstructed images at different kVs, (a_*j*_): 80kV, (b_*j*_): 100kV, (c_*j*_): 120kV, and (d_*j*_): 140 kV, for all *j* = 0 ⋯ 4. Each column has reconstructed images from different reconstruction algorithms: (X_0_): scanner, (X_1_): FDK, (X_2_): Katsevich, (X_3_): TV, and (X_4_): TF for all letters X = a, b, c or d.

Four evaluations metrics were compared on the different dosage levels of 80, 100, 120, and 140 kVs. A different x-ray source has a different effective dosage (see Table 1). We chose two slices for the evaluation process, slices 10 and 50. Figs 1 and 2 show the results for slices 10 and 50, respectively. For both figures, from left to right, each column shows the reconstructed images from the scanner, by FDK, Katsevich, TV, and TF algorithms. Each row consists of reconstructed images from different kVs: (a_*j*_)’s are from 80kV, (b_*j*_)’s are from 100 kV, (c_*j*_)’s are from 120 kV, and (d_*j*_)’s are from 140 kV, for all *j* = 0, ⋯, 4. The red circles on Fig 1 indicate specific ROIs; ROI1, ROI2, ROI3, and ROI_AIR_ for the computation of SNR and CNR. ROI 1, ROI2, and ROI3 are the interior of the small circles inside the ACR phantom. The red line in (a_0_) is the ROI for the edge spread function, used for calculating MTF. The set of interiors of the small red circles on the 50-th slice, the (a_0_) of Fig 2, is set as a ROI4 and the rest of the area except ROI4 inside of the phantom is set to the ROI_AIR_ for the computation of the SNR and CNR of ROI4. As illustrated in the Figs 1 and 2, the images from TV and TF reconstruction algorithms give clear images compared to FDK and Katsevich results.

**Fig 3:**
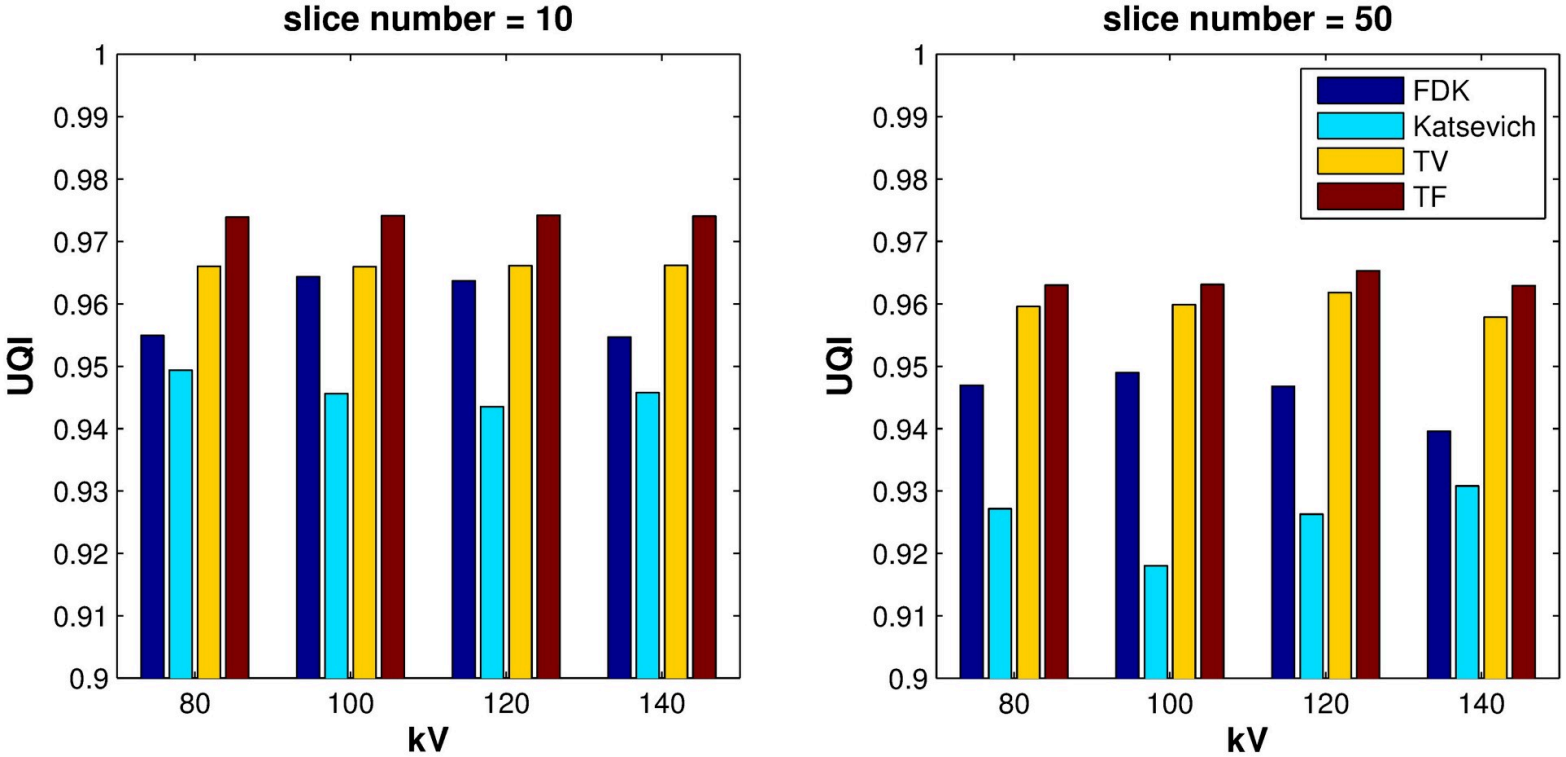
Image similarity measure: Bar plot of the UQIs of the different reconstruction algorithms over various dosage levels. (a): UQI on the 10-th slice, (b): UQI on the 50-th slice.

**Fig 4:**
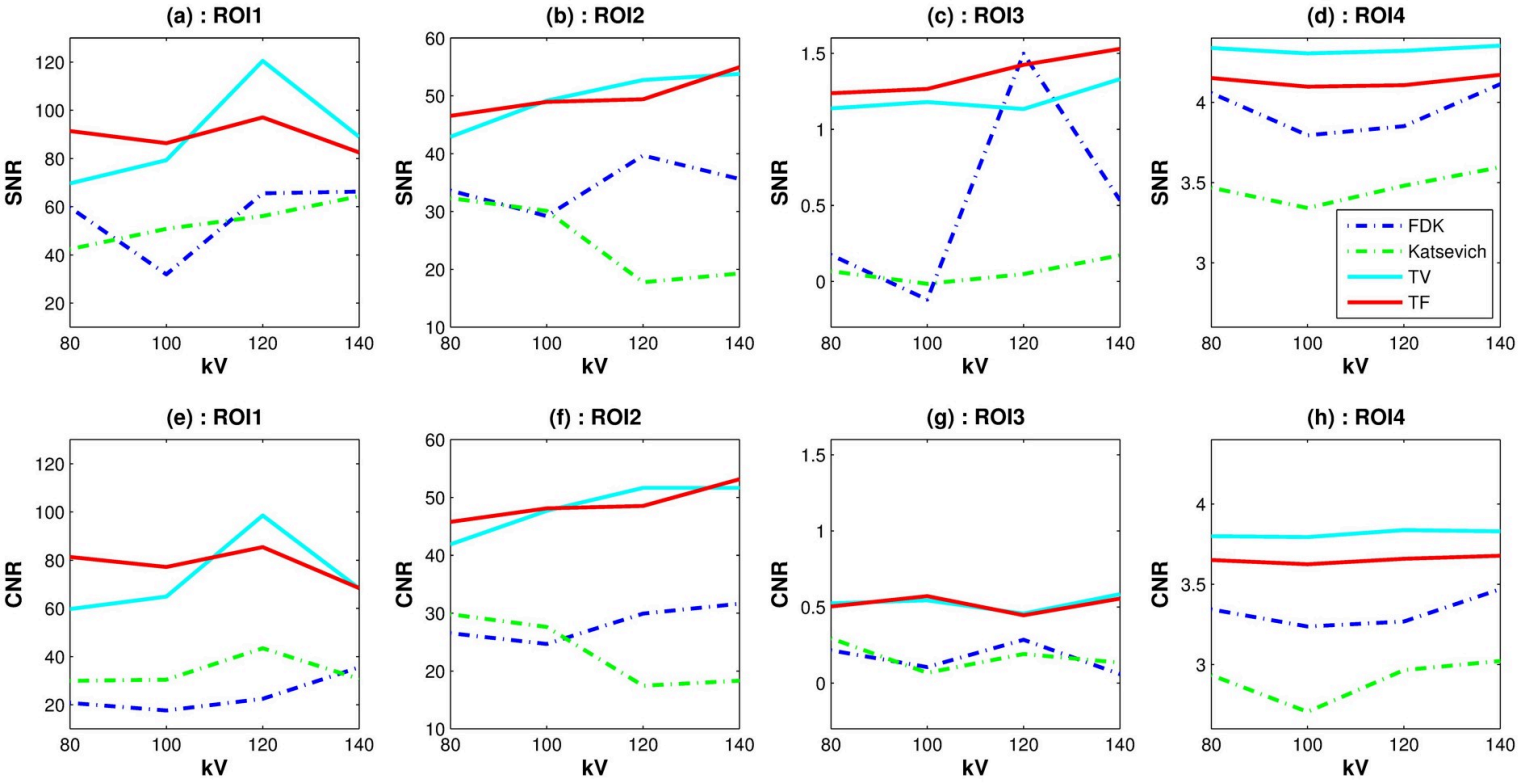
Image noise measures: Plots of SNRs(top row) and CNRs(bottom row) with the different reconstruction algorithms over various voltage levels. The *x*-axis is the dosage level in kV.

**Fig 5:**
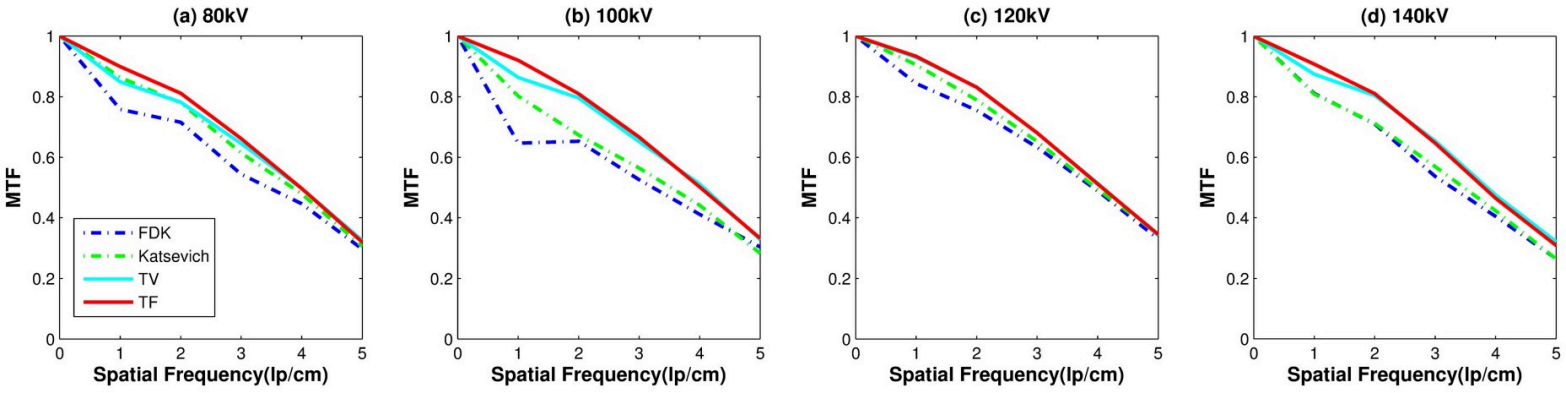
Image resolution measure: Results of MTF curves with the different reconstruction algorithms over various voltage levels. The red line on the Fig 1(a_0_) is used to compute LSF and MTF.

Quantitative evaluation results are shown in Figs 3–5. For computing the UQI, images from the scanner were treated as true. Two ROIs for the UQI are set as the whole ACR phantom on the slices 10 and 50. The left bar plot of Fig 3 shows the result of the UQI in three different algorithms of the ROI on th 10th slice. The right plot shows the UQI result of slice 50. For both plots, the TF reconstruction method achieved the closest value to 1, which means the TF reconstructed image was the most similar to the scanner results. To evaluate the noise level of the reconstructed images, Fig 4 shows the SNR and CNR results at the various dosage levels. The plots on the top row((a)-(d)) are the results of SNR over the ROI1, ROI2, ROI3, and ROI4. Note that each ROI has different y-range, since different ROI has different noise level. ROIs are defined in Figs 1 and 2. CNRs on the ROI1-ROI4 are illustrated in Fig 4(e)–4(h). TF and TV algorithms achieved the high CNR and SNR on the four ROIs at all dosage levels, except in one case: The SNR on ROI3 at 120 kV. TF and TV algorithms are comparable. TF achieved the highest value on ROI3, but TV did on ROI4.

Fig 5 shows the MTF curves of the results reconstructed by FDK, Katsevich, TV and the TF algorithm. Over all voltage levels, TF algorithms got the best resolutions than the other algorithms. But the MTF curve gives no big difference in various voltage levels.

Overall, quantitative evaluation results with various dosage show results of TF and TV are competitive.

### Evaluations with sparse-view data

**Fig 6:**
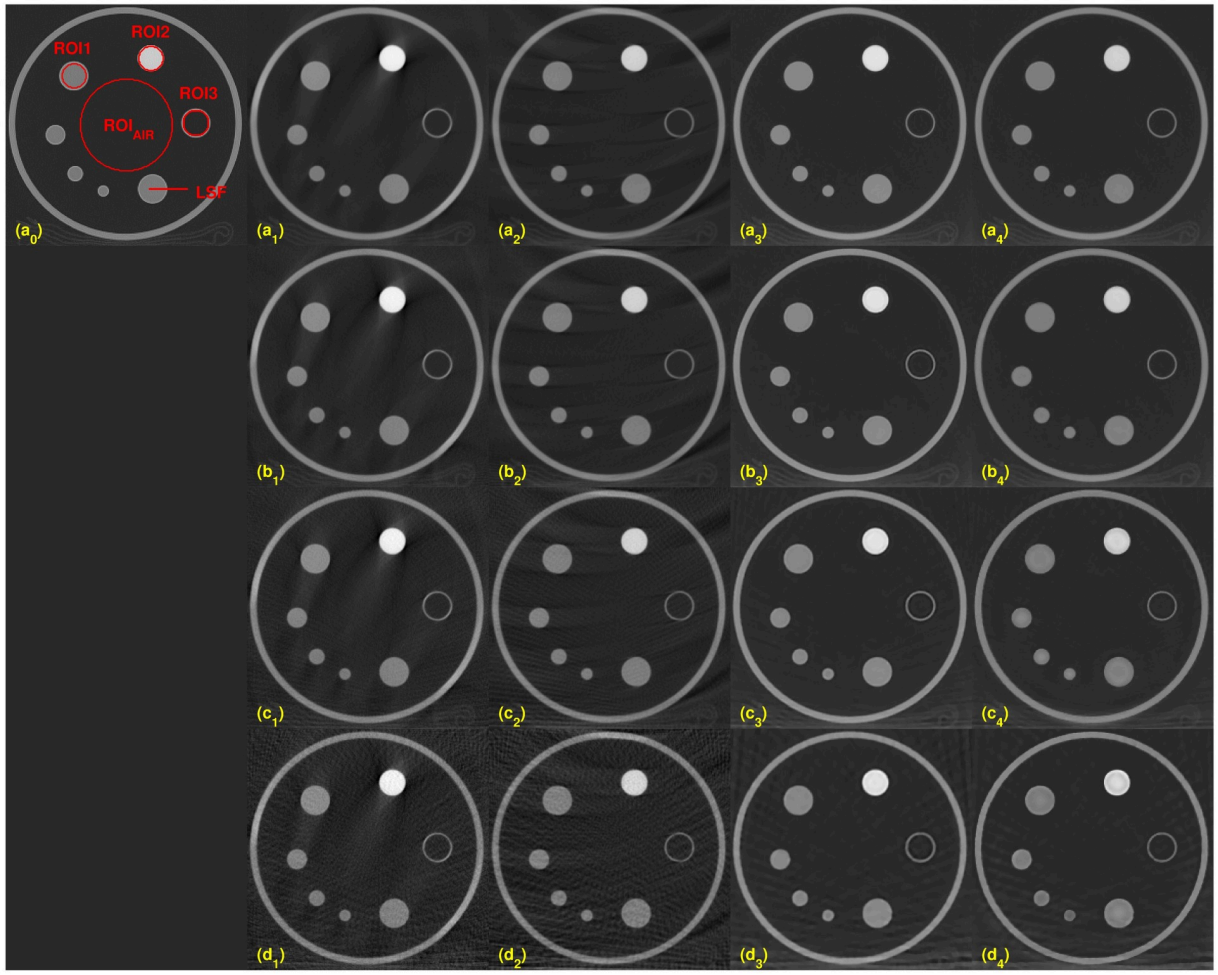
Reconstucted images with various sampling step sizes. From top to bottom, the sampling step size is set to 1, 4, 8, and 16. Each column consists of a different reconstruction algorithm, from left to right, scanner: FDK, Katsevich, TV and the TF algorithm. The image on (a_0_) shows the three ROIs, and the red line is set for the computation of LSF for MTF. ROI_AIR_, ROI of air, is defined to compute the CNR for ROI1-ROI3.

**Fig 7:**
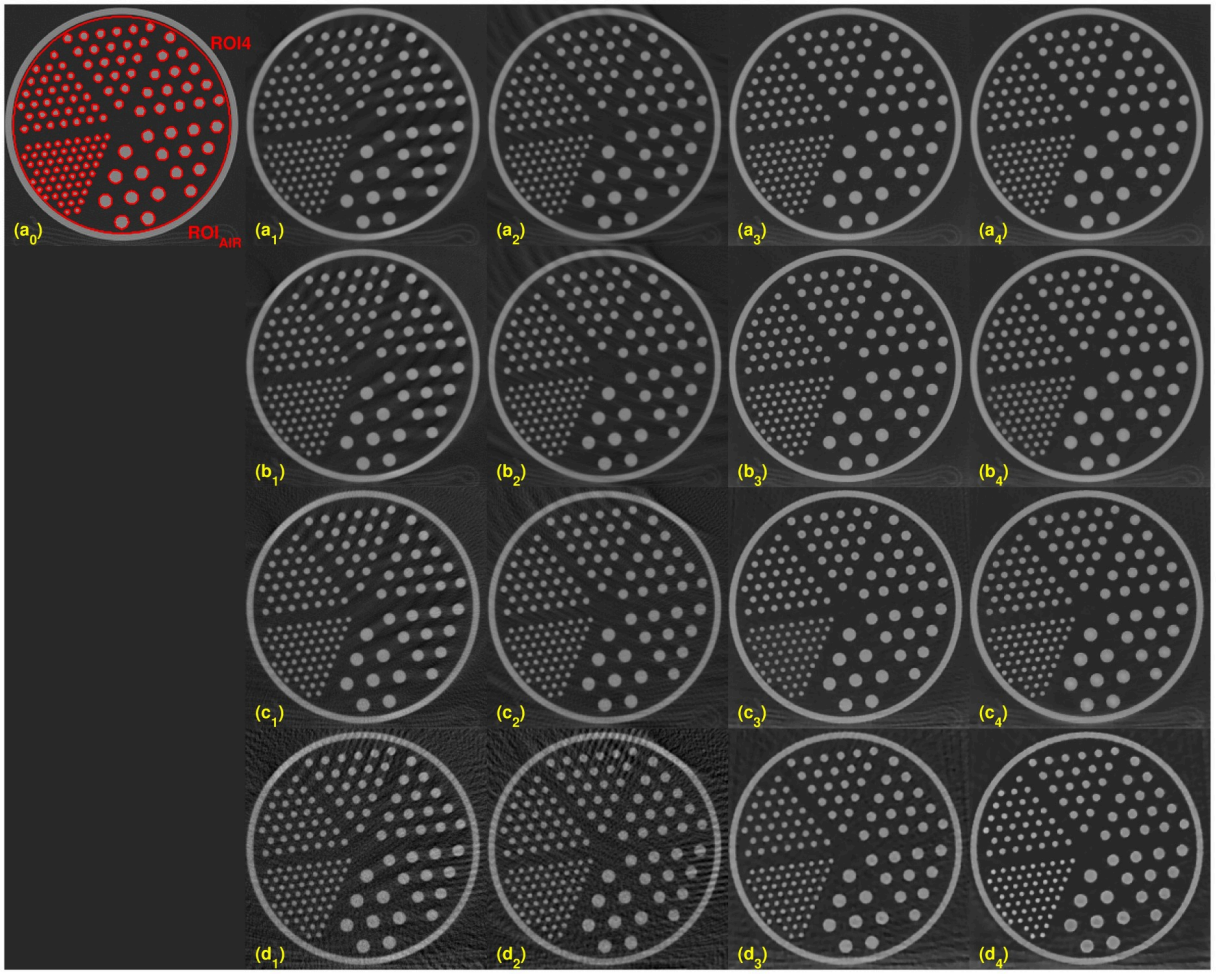
Reconstucted images with various sampling step sizes. From top to bottom, the sampling step size is set to be 1, 4, 8, and 16. Each column consists of a different reconstruction algorithm, from left to right: scanner, FDK, Katsevich, TV and the TF algorithm. The image on (a_0_) shows ROI4 and ROI_AIR_.

To evaluate with sparse-view performance, we fixed the dose level at 100kV. Images were reconstructed at four different sampling steps, 1, 4, 8, and 16. The full view data has 2304 views per 360°. Sampling step 4 was achieved by taking 576 data uniformly per 360°. Similarly, sampling steps 8 and 16 were achieved with 288 and 144 views per 360°, respectively. For sampling step 4, it is equivalent that both the rotation speed and the table movement are four times faster than those of sampling step 1. The results of the reconstruction images with different view-angles are shown in Figs 6 and 7. The images (a_0_) and (b_0_) are from the scanner on both figures. From the top to the bottom rows, images are reconstructed CT images by sampling steps 1, 4, 8, and 16. Each column shows images from a different reconstruction algorithm. From left to right, each column consists of images by scanner, FDK, Katsevich, TV and the TF algorithm. As shown in the first row, reconstruction images at sampling step 1 are streak-free for all reconstruction algorithms. However, streaks appeared on the images with FDK and Katsevich for sparse-view data. The last column of the Figs 6 and 7 showed that visually TV and TF reconstruction outperformed other two reconstruction methods. On Figs 6(a_0_) and 7(a_0_), ROI’s are defined as in the previous section. Visual comparison between TV and TF is given in the next subsection.

**Fig 8:**
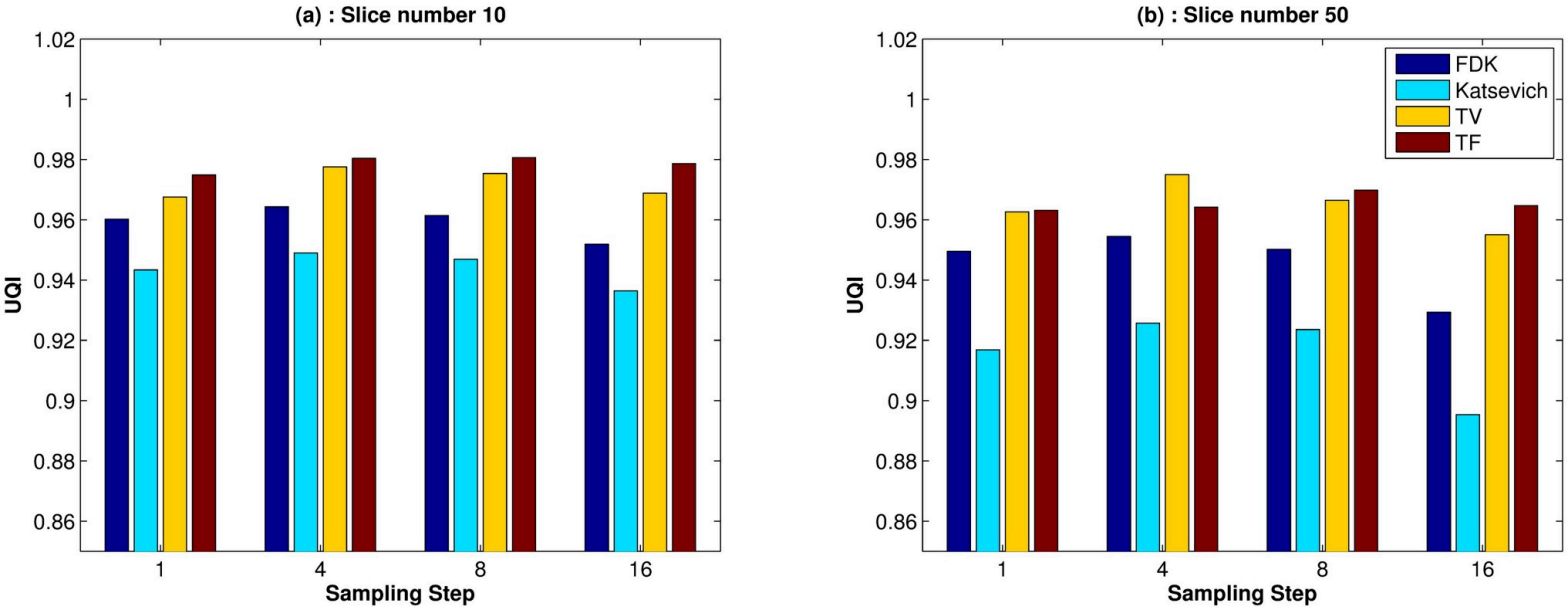
Image similarity measure: UQI results for various sampling step size. *x*- axis is the sampling step size, 1, 4, 8, and 16. *y*- axis is set as the UQI index. (a): UQI bar plot for the 10-th slice. (b): UQI bar plot for the 50th slice.

For the quantitative evaluation of similarity between the reconstructed image and the scanner image, we computed the UQI for each slices 10 and 50. The ROI for the UQI is set as the whole phantom area on a given slice. Fig 8 shows the result of UQI with various sampling step sizes. Both plots (a) and (b) show that the TF algorithm achieved the highest value except one case, which means that the image reconstructed using the TF algorithm was the most similar to the scanner image.

**Fig 9:**
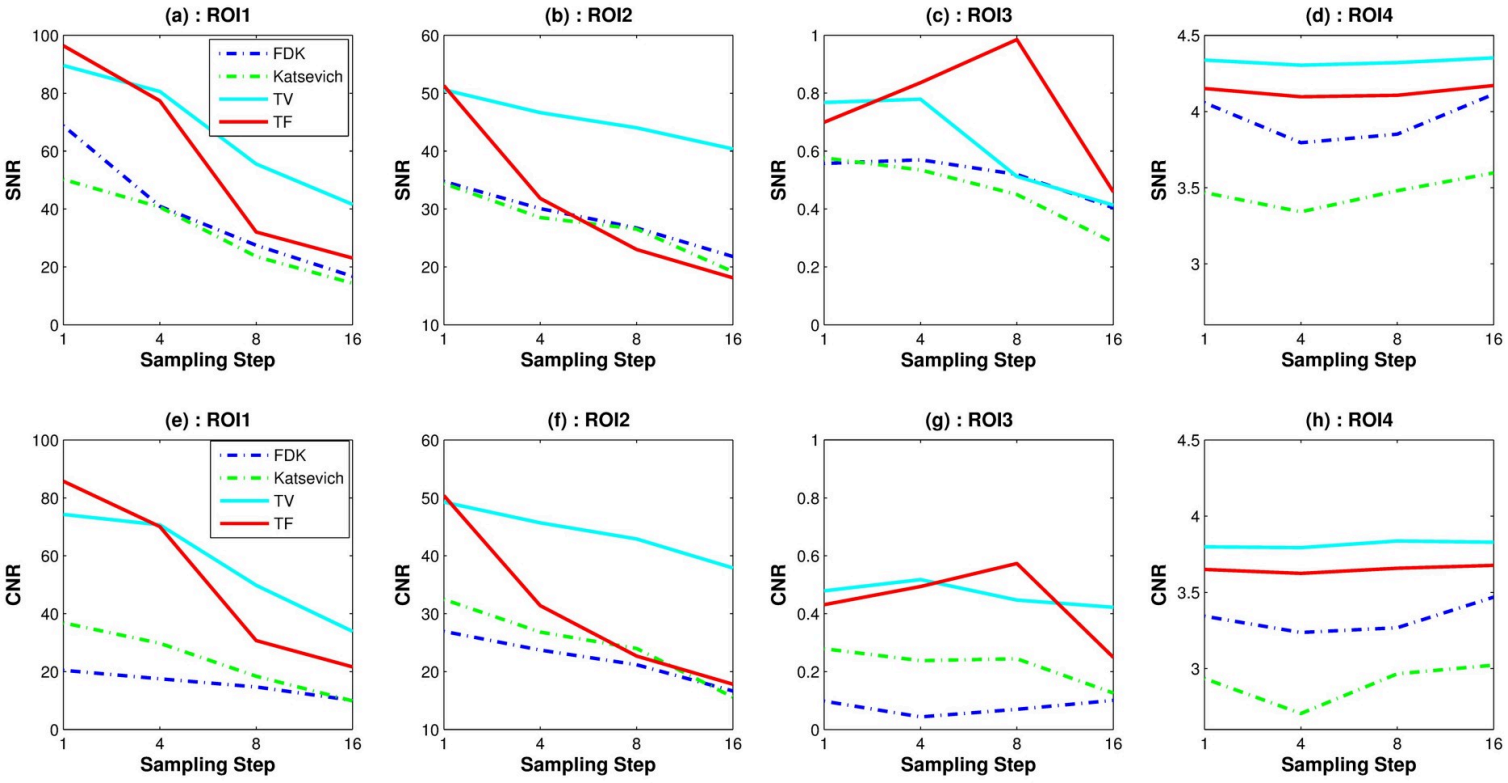
Image noise measures: SNR and CNR results for the various sampling step sizes. First row: SNR result, second row: CNR results.

**Fig 10:**
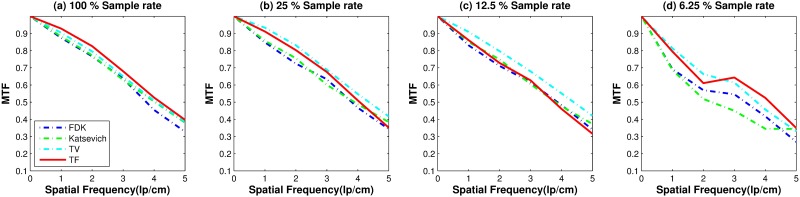
Image resolution measure: Results of MTF curves with different reconstruction algorithms over various sampling levels. The red line on the Fig 6(a_0_) is used to compute the LSF and MTF.

For the quantitative evaluation of the noise level of the reconstructed images, we computed the SNR and CNR on ROIs 1–4. Fig 9 shows the SNR and CNR results. Similar to Fig 4, each column in Fig 9 has different y-range. The first row consists of the SNR results for ROI1-ROI4. The second row is the result of the CNR of ROI1-ROI4. Both SNR and CNR indices have a similar pattern. The TF algorithm achieved the highest SNR and CNR except for a few points in ROI2 and ROI4. For the quantitative evaluation of the image resolution, Fig 10 shows MTF curves as described in the previous subsection. The LSF is computed with the ROI indicated in Fig 6(a_0_). TV and TF results achieve high resolution than other two algorithms. The TF algorithm achieved the highest MTF, especially when the fewest sample generated the highest MTF difference among other reconstructed methods.

### Comparison with TV

**Fig 11:**
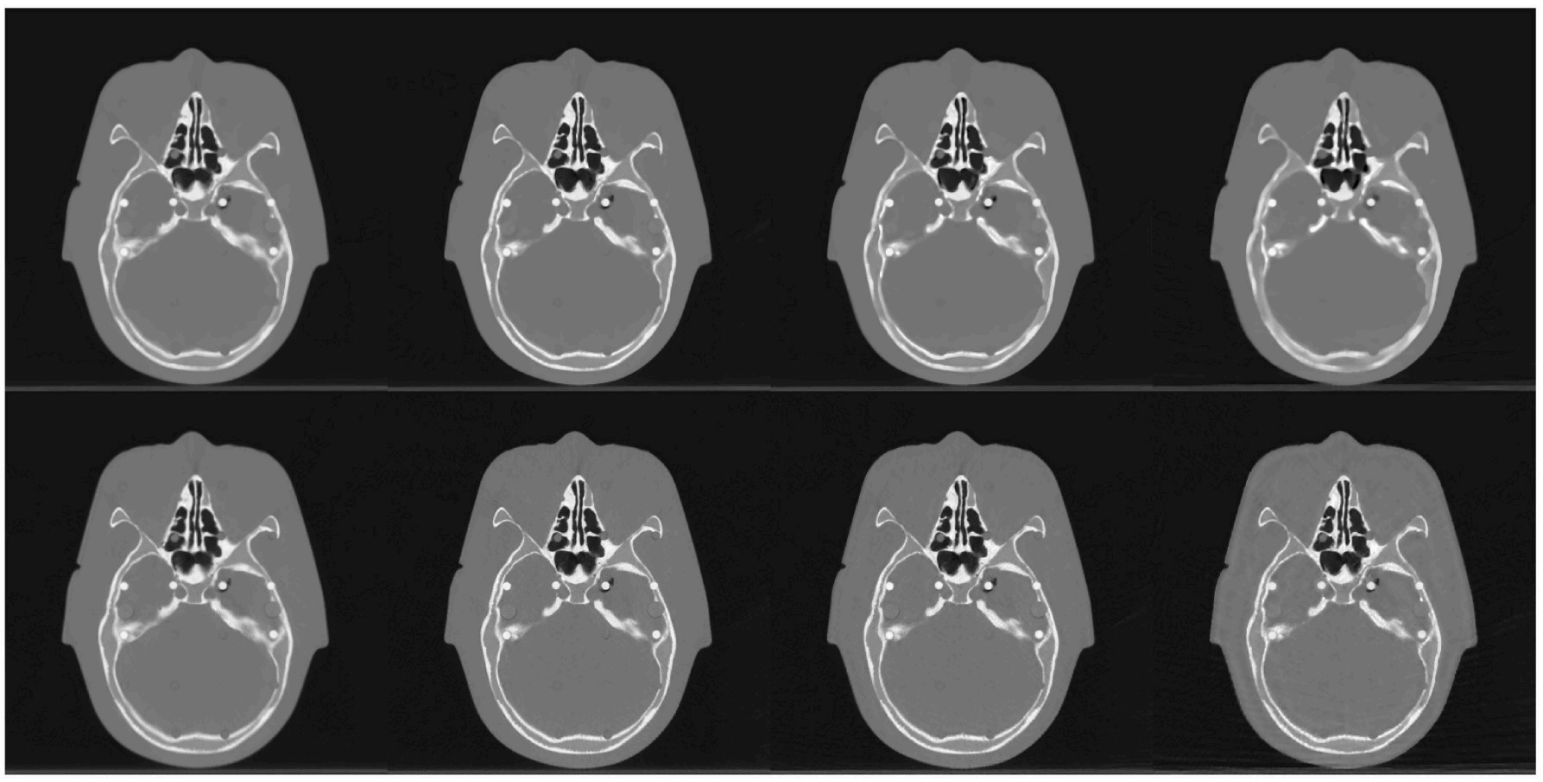
Visual quality comparison: TV(top row) and TF(bottom row). From left to right, reconstruction results by the sampling step size is set to 1, 4, 8, and 16. TV results shows more blurry effect compared to TF results.

**Fig 12:**
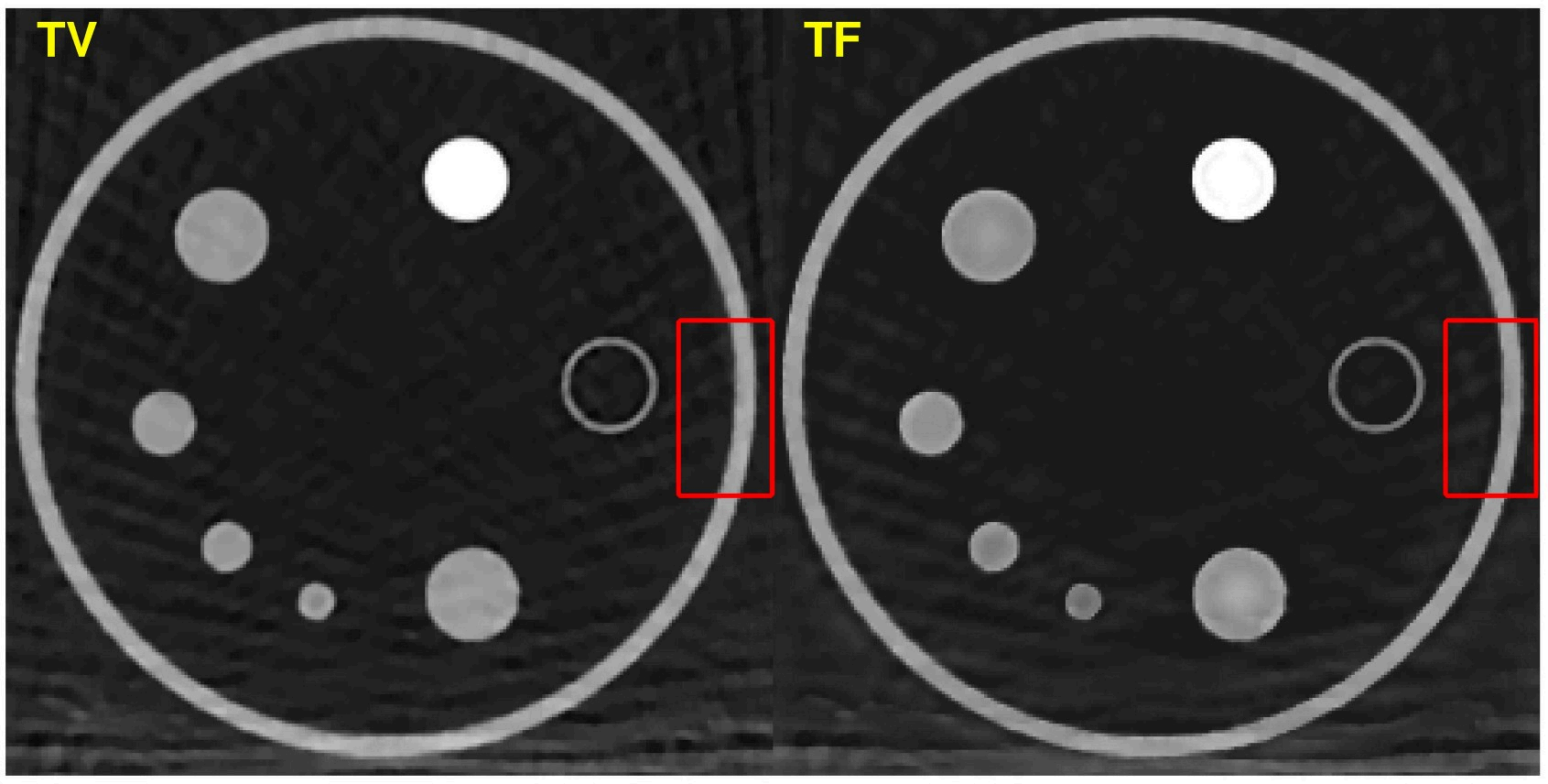
Visual quality comparison: TV(left) and TF(right). TF image has less streaking artifact. As shown in the red box, TF maintain sharpen edge.

As shown in Figs 1 ~ 7, image qualities of TF and TV are hard to compare. Each quantitative metric shows a slight superiority of TF. To show some good points of the proposed algorithm, we have tested Rando phantom data, which has more realistic and complicated structure than ACR phantom. Rando phantom is scanned and reconstructed with sparse-view as done in the previous subsection. Fig 11 shows the results by TV(top rows) and TF(bottom rows) algorithms. The sampling size 1 (which is the right column) results shows similar to each other. But as the step size increases, the TV results are more blurry but clean, while that of TF maintains sharpen edges even with a large step size. Same results can be shown in Fig 6. Fig 12 are same images from Fig 6, TV and TF reconstruction with step 16. Streaking artifacts due to partial projection data are shown less in the TF results. As indicated in red box, TF image has more sharpen edges than that of TV. Overall, we can conclude that TV and TF image qualities are similarly good, but TF has more sharpened edge and less artifacts.

One of the key factor to evaluate iterative algorithms is the reconstruction time. TV and TF elapsed times are summarized in Table 2. TF algorithm requires about 25% less time than TV algorithm.

**Table 2:** TF and TV elapsed time in seconds.

sampling step size	1	4	8	16
TF	18199	4485	1708	718
TV	23303	5674	2692	1429

## Discussion and conclusion

To summarize, we have successfully developed a GPU-based TF iterative image reconstruction algorithm for low-dose multislice helical CT, and have shown that the TF method provided improved image quality over the FDK, the Katsevich and TF algorithms when dealing with low-dose and sparse-view data, using UQI, SNR, CNR, and MTF measurements as evaluation metrics. High quality images are reconstructed by the proposed algorithm even with partial view data. TF algorithm is more computationally efficient than that of TV, because of the left-invertibility of the TF transform property [27]. Moreover, TV reconstructed images show more blurry and flattened than TF. The computational complexity of the TF algorithm is O(1), which is the cost of the x-ray transform and its adjoint per parallel thread [27].

## Supporting information

S1 Fig Data related to Fig 1. (MAT)

S2 Fig Data related to Fig 2. (MAT)

S3 Fig Data related to Fig 6. (MAT)

S4 Fig Data related to Fig 7. (MAT)

S5 Fig Data related to Fig 11. (MAT)
